# Dysregulated cholesterol metabolism in genodermatoses: implications for systemic disease and therapeutic strategies

**DOI:** 10.3389/fcell.2026.1840149

**Published:** 2026-08-27

**Authors:** Zhenzhen Xiao, Yue Kang, Rui Li, Yingjian Tan

**Affiliations:** 1 Department of Dermatology, Fuzhou First General Hospital Affiliated with Fujian Medical University, Fuzhou, China; 2 Department of Respiratory and Critical Care, Xinxiang Central Hospital, Xinxiang, Henan, China; 3 Department of Dermatology, Venereology and Allergology, Charité-Universitätsmedizin Berlin, Berlin, Germany

**Keywords:** cholesterol metabolism, gene mutation, ichthyosis, lamellar body, porokeratosis

## Abstract

Cholesterol plays a critical role in maintaining normal physiological functions, and it is particularly important in skin tissue. Dysregulation of cholesterol metabolism can lead to a spectrum of skin disorders, including ichthyosis, porokeratosis, keratosis follicularis spinulosa decalvans and related diseases. While the pathogenesis of certain genodermatoses of keratinization has been clearly linked to abnormal cholesterol metabolism, known genetic mutations account for only a subset of cases, suggesting additional cholesterol-linked modifiers, suggesting a potential involvement of cholesterol-related pathways. This review summarizes recent advances in understanding genodermatoses of keratinization associated with disrupted cholesterol metabolism and discusses their underlying molecular mechanisms. A better understanding of cholesterol dysregulation may facilitate the identification of novel diagnostic biomarkers and therapeutic targets, providing new opportunities for precision management of inherited keratinization disorders.

## 1 Introduction

Cholesterol and its metabolites are widely distributed in various human tissues, including the skin, brain, nervous system, liver, and kidneys, and play essential roles in maintaining normal physiological functions (Nowowiejska et al., 2023). Cholesterol homeostasis in the body is maintained through a highly complex process involving both *de novo* biosynthesis and dietary uptake, while cholesterol catabolism contributes to the synthesis of biologically important molecules such as vitamin D3 and steroid hormones (Sanabria-de la Torre et al., 2025). In the skin, proper cholesterol metabolism is crucial for the maintenance of epidermal homeostasis and barrier function (Rischke et al., 2024).

Mutations in genes regulating cholesterol biosynthesis, degradation, modification, and transport can lead to a spectrum of dermatological disorders, including ichthyosis, porokeratosis, keratosis follicularis spinulosa decalvans and related diseases (Berdyshev, 2024). Genodermatoses of keratinization comprise a heterogeneous group of inherited skin disorders characterized primarily by dry skin, hyperkeratosis, and scaling. However, therapeutic outcomes for these skin diseases are often unsatisfactory, largely due to the incomplete understanding of their underlying pathogenic mechanisms.

With the identification of causative genes associated with multiple genodermatoses of keratinization, the critical involvement of cholesterol metabolism in their pathogenesis has become increasingly evident. This review summarizes recent advances in understanding how gene mutations affecting cholesterol metabolic pathways contribute to the development and progression of genodermatoses of keratinization (Tables 1, 2). This review primarily focuses on inherited keratinization disorders and epidermal barrier abnormalities directly associated with cholesterol and sterol dysregulation in the epidermis. Systemic lipid disorders with secondary or limited cutaneous manifestations, including xanthomatous and intracellular cholesterol trafficking disorders, are not comprehensively discussed. This narrative review was conducted through a literature search in PubMed, Web of Science, and Scopus databases up to January 2026. Search terms included combinations of “cholesterol metabolism,” “genodermatoses,” “ichthyosis,” “keratinization disorders,” “mevalonate pathway,” “ABCA12,” and related terms. Articles published in English were primarily included. Relevant original studies, reviews, and genetic reports were screened for inclusion based on relevance to cholesterol-associated epidermal disorders.

**TABLE 1:** Abbreviation of genes, enzymes and diseases.

Abbreviation	Full name	Abbreviation	Full name
HMG-CoA	3-Hydroxy-3-methylglutaryl coenzyme A	ERAD	Endoplasmic reticulum–associated degradation
HMGCR	3-Hydroxy-3-methylglutaryl coenzyme A reductase	INSIG	Insulin-induced gene
SREBP	Sterol regulatory element-binding protein	SULT2B1	Hydroxysteroid sulfotransferase 2B1
SCAP	SREBP cleavage-activating protein	STS	Steroid sulfatase
LDLR	Low-density lipoprotein receptor	CHILD	Congenital hemidysplasia with ichthyosiform naevus and limb defects
S1P	Site-1 protease	NSDHL	NAD(P)H steroid dehydrogenase-like
S2P	Site-2 protease	CDPX2	Chondrodysplasia punctata type 2
SREs	Sterol regulatory elements	EBP	Emopamil-binding protein
XLI	X-linked ichthyosis	MSD	Multiple sulfatase deficiency
SUMF1	Sulfatase-modifying factor 1	IFAP	Ichthyosis follicularis with alopecia and photophobia
MBTPS2	Membrane-bound transcription factor peptidase, site 2	SREBF1	Sterol regulatory element binding transcription factor 1
HI	Harlequin ichthyosis	ARCI	Autosomal recessive congenital ichthyosis
ABCA12	ATP-binding cassette subfamily A member 12	LI	Lamellar ichthyosis
CIE	Congenital ichthyosiform erythroderma	PPKCA2	Palmoplantar keratoderma–congenital alopecia syndrome type 2
LSS	Lanosterol synthase	CRISPR	Clustered regularly interspaced short palindromic repeats
PK	Porokeratosis	KFSD	Keratosis follicularis spinulosa decalvans
CST6	Cystatin 6	MVK	Mevalonate kinase
PMVK	Phosphomevalonate kinase	MVD	Mevalonate diphosphate decarboxylase
FDPS	Farnesyl diphosphate synthase	FDFT1(SQS)	Farnesyl-diphosphate farnesyltransferase 1
IDI	Isopentenyl-diphosphate delta-isomerase	FDPS	Farnesyl diphosphate synthase
SQS(FDFT1)	Squalene synthase	SQLE	Squalene epoxidase
DHCR7	7-Dehydrocholesterol reductase	IPP	Isopentenyl pyrophosphate
Mevalonate-5-P	Mevalonate-5-phosphate	DMAPP	Dimethylallyl pyrophosphate
Mevalonate-5-PP	Mevalonate-5-diphosphate	GPP	Geranyl pyrophosphate
FPP	Farnesyl pyrophosphate	GGPS1	Geranylgeranyl diphosphate synthase 1
FF-MAS	Follicular fluid Meiosis-Activating sterol	CYP51A1	Cytochrome P450 family 51 subfamily A member 1
T-MAS	Testis Meiosis-Activating sterol	TM7SF2	Transmembrane 7 superfamily member 2
MSMO1	Methylsterol monooxygenase 1	SC5D	Sterol-C5-desaturase
HSD17B7	Hydroxysteroid 17-beta dehydrogenase 7	7-DHC	7-Dehydrocholesterol

**TABLE 2:** Summary of Disease-Causing Genes, Clinical Manifestations and Immunologic features.

Disease	Causing gene	Metabolic pathway	Clinical manifestations	Immunologic features
CHILD syndrome	*NSDHL* (OMIM: 300275)	Cholesterol transport and traffic	Unilateral ichthyosiform erythematous plaques sharply limited to one body side, often with ipsilateral limb hypoplasia or absence and occasional internal organ involvement	Mutations in *NSDHL* cause toxic sterol accumulation, inducing oxidative stress and inflammatory signaling in keratinocytes
CDPX2	*EBP*(OMIM: 300205)	Distal sterol processing defects	Ichthyosiform lesions along Blaschko lines, skeletal abnormalities, cataracts, patchy alopecia, and pigmentary changes developing after neonatal erythroderma	Mutations in *EBP* lead to sterol intermediate accumulation, promoting oxidative stress and inflammatory cytokine production
X-linked ichthyosis	*STS*(OMIM: 300747)	Cholesterol transport and traffic	Generalized dark polygonal scales on trunk and extensor surfaces with sparing of flexures; corneal opacities and cryptorchidism may occur	Loss of *STS* causes cholesterol sulfate accumulation, altering keratinocyte differentiation and promoting mild inflammatory signaling
Multiple sulfatase deficiency	*SUMF1*(OMIM: 607939)	Cholesterol transport and traffic	Generalized ichthyosis accompanied by progressive neurodegeneration, developmental delay, skeletal abnormalities, and hepatosplenomegaly	Mutations in *SUMF1* impair sulfatase activation, leading to lysosomal storage stress and inflammatory responses
IFAP syndrome	*MBTPS2*(OMIM: 300294) *SREBF1* (OMIM: 184756)	SREBP-SCAP pathway	Follicular hyperkeratosis, congenital alopecia, and severe photophobia with possible neurological impairment and ocular abnormalities	Mutations in SREBP-SCAP pathway disrupt sterol regulatory signaling and induce ER stress–related inflammation
Harlequin ichthyosis	*ABCA12*(OMIM: 607800)	Cholesterol transport and traffic	Severe congenital ichthyosis with thick armor-like hyperkeratotic plates, deep fissures, ectropion, eclabium, and limb contractures; affected neonates often experience dehydration and infection	Mutations in *ABCA12* impair lipid transport in lamellar granules, causing barrier failure and secondary activation of inflammatory cytokine signaling
Lamellar ichthyosis	*ABCA12*(OMIM: 607800), *SULT2B1*(OMIM: 604125), *TGM1*(OMIM: 190195), *CYP4F22*(OMIM: 611495), *ALOXE3*(OMIM: 607206), *ALOX12B*(OMIM: 603741), *NIPAL4*(OMIM: 609383), *SDR9C7*(OMIM: 609769), *CERS3*(OMIM: 615276), *PNPLA1*(OMIM: 612121)	Cholesterol transport and traffic	Large dark plate-like scales covering the body, often present after a collodion membrane at birth; ectropion and palmoplantar keratoderma may occur	Mutations in genes such as *TGM1* disrupt cornified envelope formation, leading to barrier dysfunction and increased epidermal inflammatory signaling
CIE	*ABCA12*(OMIM: 607800), *TGM1*(OMIM: 190195), *CYP4F22*(OMIM: 611495), *ALOXE3*(OMIM: 607206), *ALOX12B*(OMIM: 603741), *NIPAL4*(OMIM: 609383), *SDR9C7*(OMIM: 609769), *CERS3*(OMIM:615276), *PNPLA1*(OMIM: 612121), *LIPN*(OMIM: 613924)	Cholesterol transport and traffic	Generalized erythroderma with fine white scaling from birth, frequently associated with a collodion membrane and variable palmoplantar keratoderma	Defects in genes such as *ALOX12B* impair epidermal lipid metabolism, promoting barrier defects and chronic cutaneous inflammation
PPKCA2	*LSS*(OMIM: 600909)	Distal sterol processing defects	Diffuse palmoplantar keratoderma accompanied by congenital alopecia and progressive hyperkeratosis of palms and soles	Mutations in *LSS* disturb sterol regulatory and ER-stress pathways, which may enhance inflammatory responses in keratinocytes
Porokeratosis	*MVK*(MIM: 251170), *PMVK*(MIM: 607622), *MVD* (MIM: 603236), *FDPS*(MIM: 134629), *FDFT1* (MIM: 184420)	Mevalonate pathway	Annular keratotic plaques with elevated ridged borders and central atrophy, histologically characterized by cornoid lamella	Mutations in mevalonate pathway genes such as MVK lead to metabolic stress and activation of inflammatory signaling in keratinocytes
KFSD	*MBTPS2*(OMIM: 300294), *CST6*(OMIM: 601891)	SREBP-SCAP pathway	Follicular hyperkeratosis with progressive scarring alopecia, photophobia, and keratotic papules on face and trunk	Mutations in *MBTPS2* or *CST6* impair sterol regulation and ER stress responses, promoting inflammatory signaling and follicular damage

## 2 Overview of cholesterol metabolic pathways

Cholesterol is a multifunctional lipid that plays essential roles in maintaining cellular structure, signaling, and overall tissue homeostasis (Cheng et al., 2018). In the epidermis, cholesterol is a major component of the lipid matrix in the stratum corneum, along with free fatty acids and ceramides, contributing to the barrier function that prevents water loss and protects against environmental insults. Beyond its structural role, cholesterol serves as a precursor for steroid hormones, bile acids, and vitamin D, which are critical for systemic physiological processes (Prabhu et al., 2016). Dysregulation of cholesterol metabolism in keratinocytes not only affects epidermal barrier integrity but can also lead to abnormal keratinocyte differentiation, desquamation defects, and the pathogenesis of genodermatoses of keratinization, such as ichthyosis, porokeratosis, and keratosis follicularis spinulosa decalvans (Fischer and Bourrat, 2020). Cholesterol homeostasis in the skin relies on a tightly coordinated balance among *de novo* biosynthesis, dietary uptake, intracellular transport, enzymatic modification, and catabolism, which collectively ensure adequate cholesterol availability in the stratum corneum and maintain epidermal homeostasis (Elias et al., 2004; Tokoro et al., 2025).

### 2.1 *De novo* cholesterol biosynthesis and the SREBP–SCAP pathway

**FIGURE 1:**
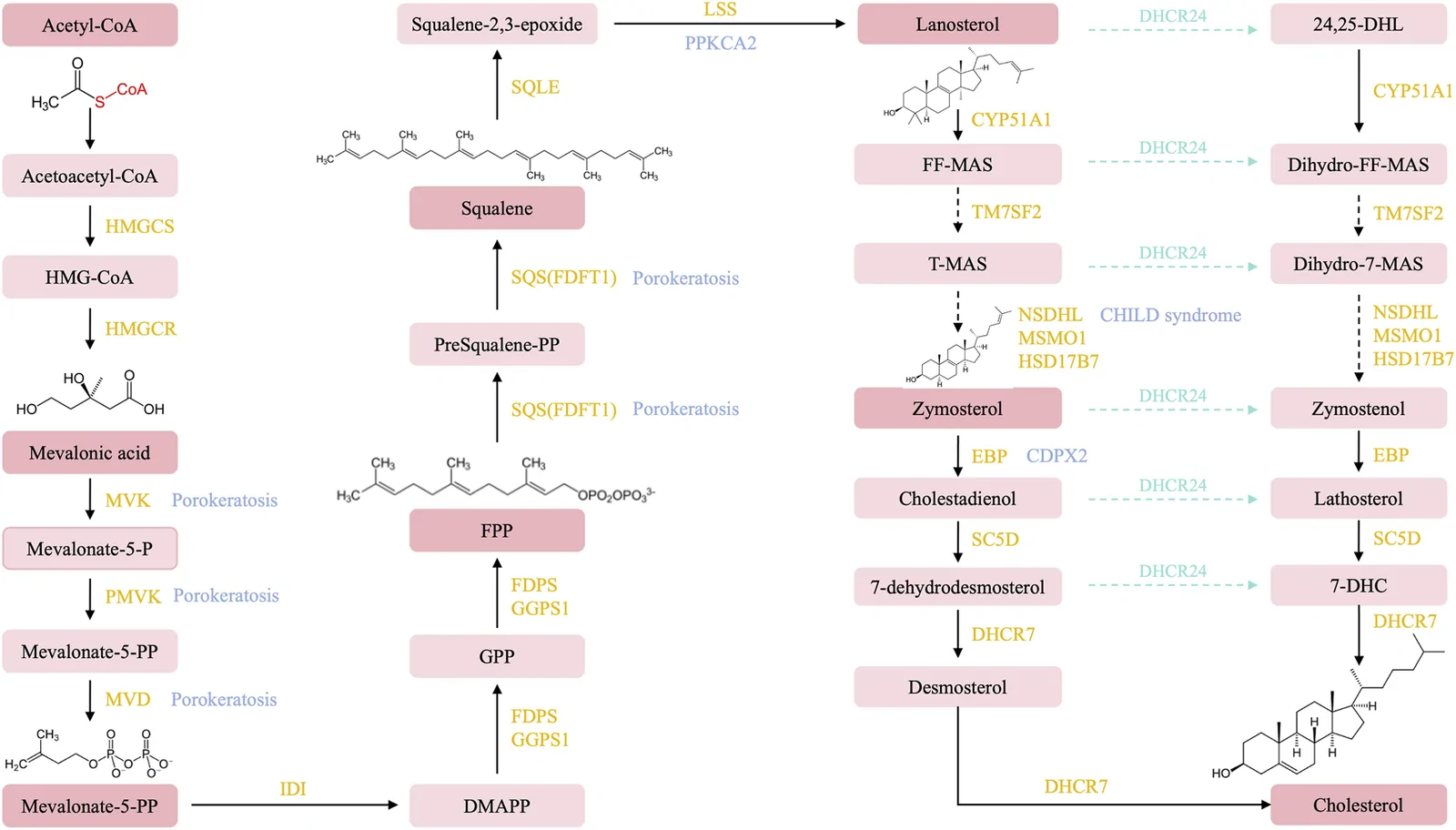
Cholesterol *de novo* biosynthesis pathway and associated genetic skin disorders. This schematic illustrates the *de novo* cholesterol biosynthesis pathway, highlighting key metabolic intermediates and the corresponding catalytic enzymes (yellow labels). Genetic mutations affecting several enzymes in this pathway are associated with specific inherited skin disorders, indicated in blue. DHCR24 is highlighted in cyan to indicate its unique role as a branch-point enzyme connecting the Bloch and Kandutsch–Russell pathways. The figure summarizes the relationship between cholesterol metabolism and dermatological diseases. The illustration was created using the Powerpoint.

*De novo* cholesterol biosynthesis (Figure 1) is a complex enzymatic process that begins with acetyl-CoA and involves over 30 sequential enzymatic reactions (Zhang et al., 2017; Shimano and Sato, 2017). Early steps include the condensation of acetyl-CoA to form 3-hydroxy-3-methylglutaryl coenzyme A (HMG-CoA), which is subsequently converted to mevalonate by 3-hydroxy-3-methylglutaryl coenzyme A reductase (HMGCR). Mevalonate serves as a precursor for isoprenoid intermediates that are further converted into squalene, lanosterol, and ultimately cholesterol (Cheng et al., 2018). These reactions occur primarily in the endoplasmic reticulum (ER) membrane and are tightly regulated to maintain intracellular cholesterol levels (Elias et al., 2012).

**FIGURE 2:**
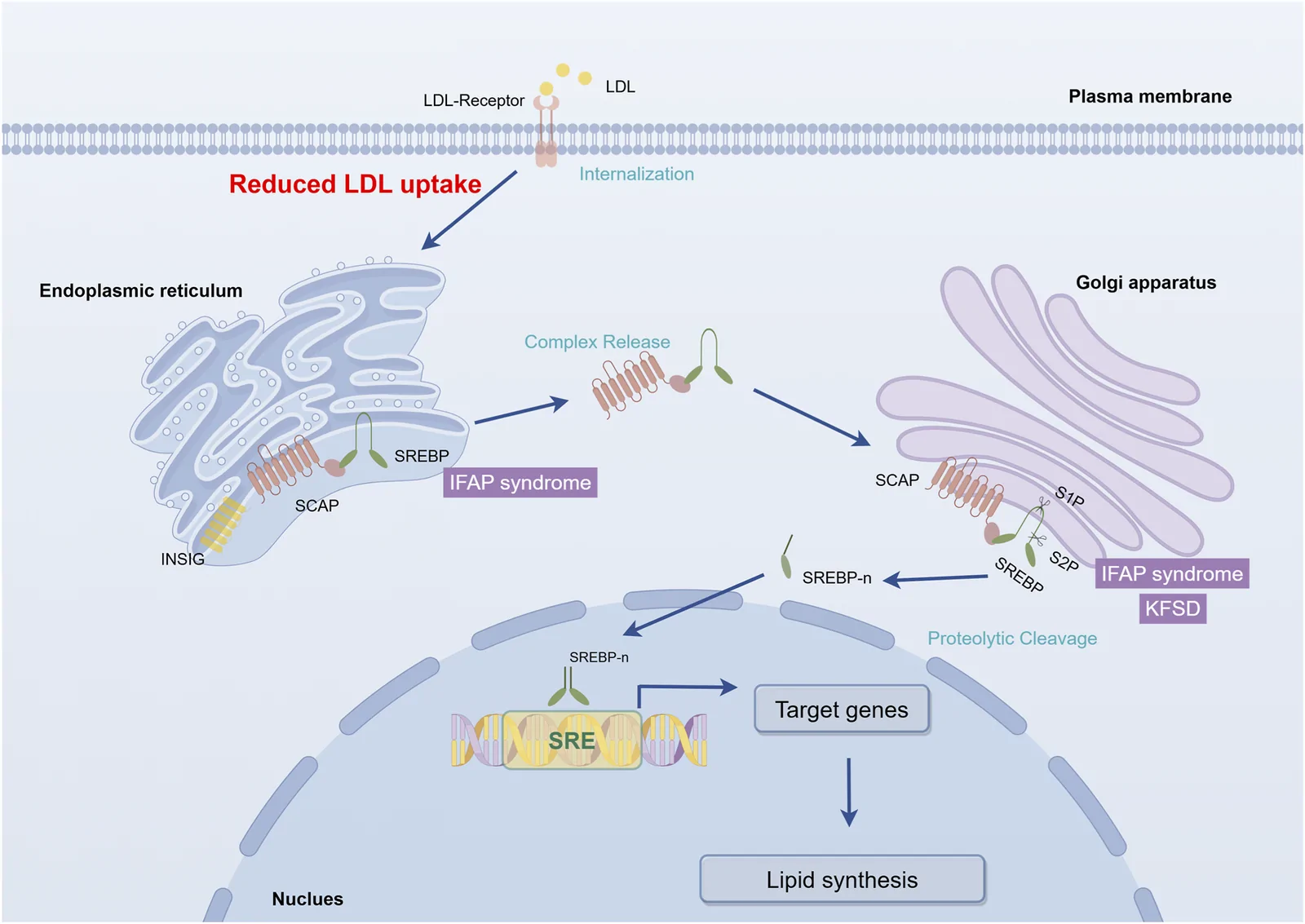
SREBP-SCAP activation pathway triggered by reduced LDL uptake. This diagram depicts how decreased LDL uptake stimulates the SREBP-SCAP complex to translocate from the endoplasmic reticulum to the Golgi and subsequently to the nucleus, activating cholesterol biosynthesis genes. The illustration was generated using the Figdraw scientific drawing platform.

A central regulatory mechanism in cholesterol biosynthesis is the SREBP–SCAP (sterol regulatory element-binding protein–SREBP cleavage-activating protein) pathway (Figure 2). SREBPs are membrane-bound transcription factors that regulate genes encoding enzymes involved in cholesterol biosynthesis and uptake, including HMGCR, HMGCS (HMG-CoA synthase), and low-density lipoprotein receptor (LDLR) (Li et al., 2023). In basal conditions with sufficient cholesterol, SREBPs are retained in the ER membrane through association with SCAP and insulin-induced gene (INSIG) proteins. Upon cholesterol depletion, the SREBP–SCAP complex is transported to the Golgi apparatus, where sequential cleavage by site-1 protease (S1P) and site-2 protease (S2P) releases the active N-terminal fragment (Huang et al., 2025). This fragment translocates into the nucleus, binds to sterol regulatory elements (SREs), and promotes transcription of target genes.

The SREBP–SCAP pathway not only regulates cholesterol synthesis but also interacts with fatty acid metabolism (Li et al., 2023). For instance, SREBP1 primarily regulates fatty acid biosynthesis, while SREBP2 preferentially controls cholesterol metabolism; however, both can cross-regulate pathways in keratinocytes, linking sterol and lipid metabolism to epidermal differentiation. Experimental studies have shown that disruption of SREBP processing in keratinocytes can lead to reduced cholesterol synthesis, impaired lipid organization in the stratum corneum, and abnormal desquamation (Li et al., 2023). This highlights the importance of SREBP–SCAP signaling in maintaining skin barrier function and suggests that mutations affecting this pathway may contribute to keratinization disorders.

### 2.2 HMGCR degradation pathway

HMGCR is the rate-limiting enzyme in cholesterol biosynthesis and is subject to precise post-translational regulation (Lin et al., 2023). One key mechanism is sterol-accelerated HMGCR degradation through the endoplasmic reticulum–associated degradation (ERAD) pathway (Eberlé et al., 2004). When intracellular sterol levels rise, HMGCR undergoes conformational changes that enable its recognition by E3 ubiquitin ligases, leading to ubiquitination and subsequent proteasomal degradation. INSIG proteins facilitate this process by stabilizing the interaction between HMGCR and ubiquitin ligases. This feedback mechanism prevents excessive cholesterol accumulation and maintains metabolic homeostasis (Jo and DeBose-Boyd, 2022).

In keratinocytes, proper regulation of HMGCR is particularly critical. Excess sterol intermediates can be cytotoxic, disrupting lamellar body formation and impairing the lipid barrier (Nowowiejska et al., 2023). Conversely, insufficient cholesterol synthesis due to premature HMGCR degradation can lead to a deficiency of cholesterol in the stratum corneum, weakening barrier integrity and predisposing the skin to dryness, scaling, and increased susceptibility to environmental insults (Sanabria-de la Torre et al., 2025). Several studies of genetic keratinization disorders have identified mutations or regulatory defects that impair HMGCR stability or activity, supporting the notion that precise control of cholesterol biosynthesis is essential for epidermal homeostasis.

### 2.3 Cholesterol transport from the basal layer to the stratum corneum

**FIGURE 3:**
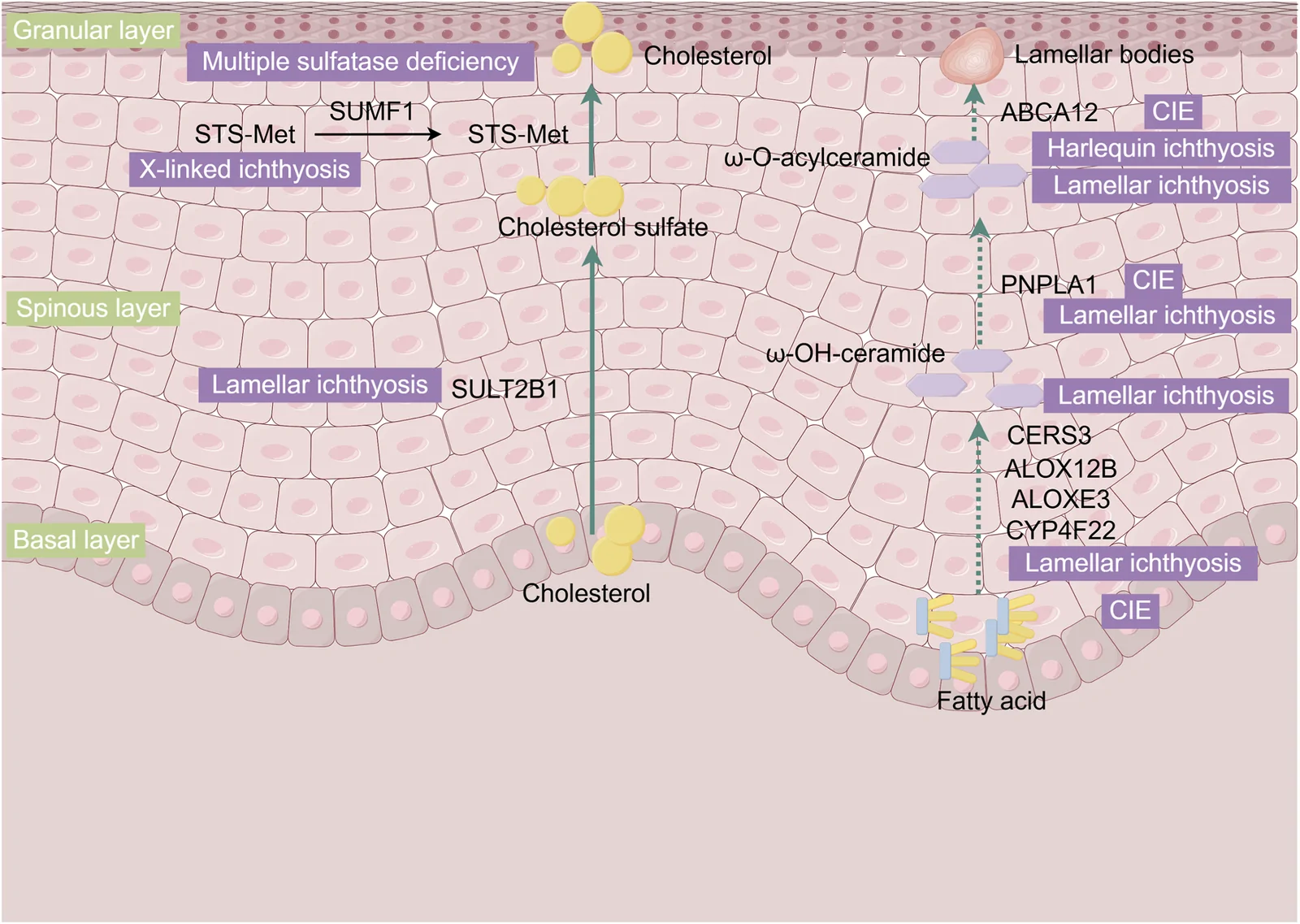
Cholesterol transport in the epidermis. This schematic summarizes the major pathways of cholesterol transport in the epidermis, emphasizing the movement of cholesterol esters and ceramides between cellular compartments. STS-Met represents steroid sulfatase-mediated cholesterol sulfate metabolism. The diagram highlights lipid trafficking critical for skin barrier function. Illustration created using the Figdraw scientific drawing platform.

Cholesterol transport within the epidermis is a highly coordinated process that ensures proper distribution of cholesterol to the stratum corneum, where it contributes to barrier function and keratinocyte desquamation (Figure 3). In differentiating keratinocytes of the suprabasal layers, cholesterol is converted to cholesterol sulfate by hydroxysteroid sulfotransferase 2B1 (SULT2B1) (Mueller et al., 2015). As keratinocytes differentiate and migrate upward through the spinous and granular layers, cholesterol sulfate is desulfated by steroid sulfatase (STS) to regenerate free cholesterol (Kwon et al., 2025).

Cholesterol sulfate has multiple critical functions in the epidermis. It is a known serine protease inhibitor that modulates the activity of corneodesmosomal proteases, including kallikreins and trypsin-like enzymes (Schl et al., 2020b). By inhibiting these proteases, cholesterol sulfate regulates corneodesmosome degradation and controls the rate of desquamation, maintaining stratum corneum integrity (Heinz et al., 2017). Additionally, the sulfate groups of cholesterol sulfate interact with intercellular calcium ions, stabilizing connections between adjacent corneocytes and reinforcing the structural cohesion of the epidermis. This calcium-mediated stabilization supports keratinocyte differentiation, adhesion, and orderly shedding (Elias et al., 2004).

Beyond desquamation, cholesterol and its derivatives are essential for lamellar body formation in the granular layer (De Greef and Baeck, 2024). Lamellar bodies are specialized organelles that deliver lipids—including cholesterol, free fatty acids, and ceramides—to the extracellular space in the stratum corneum, forming the lipid bilayers that underlie barrier function (Elias et al., 2004). Disruption of SULT2B1 or STS activity can result in abnormal cholesterol sulfate accumulation or depletion, leading to impaired lamellar body formation, defective barrier function, delayed corneodesmosome degradation, and pathological scaling, as observed in genodermatoses of keratinization (Heinz et al., 2017; Elias et al., 2013). These findings underscore the importance of tightly regulated cholesterol modification and transport in maintaining both the structural and functional integrity of the epidermis.

Collectively, the orchestration of cholesterol biosynthesis, regulation via SREBP–SCAP signaling, feedback degradation through HMGCR pathways, and epidermal transport and modification are all crucial for normal skin physiology (Shimano and Sato, 2017). Disruptions at any of these steps can compromise barrier integrity, alter keratinocyte differentiation, and contribute to the pathogenesis of inherited keratinization disorders. Understanding these pathways provides a foundation for elucidating the molecular mechanisms underlying genodermatoses of keratinization and highlights potential therapeutic targets, including modulation of cholesterol synthesis, transport, and metabolism.

## 3 Mendelian disorders along the pathway

### 3.1 Cholesterol de novo biosynthesis defects

#### 3.1.1 Mevalonate pathway

The mevalonate pathway is a highly conserved metabolic cascade responsible for the synthesis of cholesterol and non-sterol isoprenoids from acetyl-CoA. In addition to generating cholesterol, it produces key intermediates required for protein prenylation, membrane integrity, and intracellular signaling. Dysregulation of this pathway affects both structural lipid homeostasis and inflammatory signaling networks. Porokeratosis (PK) is an uncommon disorder of keratinization characterized by the presence of annular plaques with an atrophic center and a raised keratotic border corresponding histologically to the cornoid lamella (Muradia et al., 2023). Genetic studies indicate that PK can arise from mutations in genes regulating keratinocyte differentiation and epidermal lipid metabolism, including *MVK* (Mevalonate kinase, OMIM: 251170), *PMVK* (Phosphomevalonate kinase, OMIM: 607622), *MVD* (Mevalonate decarboxylase, OMIM: 603236), *FDPS* (Farnesyl diphosphate synthase, OMIM: 134629), and *FDFT1* (Farnesyl-diphosphate farnesyltransferase 1, OMIM: 184420) which are related to the mevalonate pathway (Saito et al., 2024; Zhu et al., 2019). Importantly, certain forms of PK carry a risk of malignant transformation, most commonly to squamous cell carcinoma, and it is therefore considered by some authors to represent a premalignant condition (Kostopoulos-Kanitakis and Kanitakis, 2024). Management of PK remains challenging and varies according to the clinical subtype and extent of lesions. Conventional therapies have included topical keratolytics, retinoids, cryotherapy, and laser-based approaches with variable efficacy. More recently, pathogenesis-based treatments have emerged. Topical formulations combining statins and cholesterol have shown promising results by targeting abnormalities in the mevalonate pathway implicated in PK (Atzmony et al., 2019; Santa Lucia et al., 2023). In addition, emerging evidence suggests that Janus kinase (JAK) inhibitors may represent a potential therapeutic option in selected cases, reflecting increasing interest in targeted immunomodulatory strategies for this disorder (Zhang et al., 2025).

#### 3.1.2 NSDHL is involved in removal of C4 methyl groups during cholesterol biosynthesis

##### 3.1.2.1 Congenital hemidysplasia with ichthyosiform naevus and limb defects (CHILD) syndrome

Congenital hemidysplasia with ichthyosiform naevus and limb defectssyndrome is a rare X-linked dominant, male-lethal multisystem disorder (Yoneda, 2016; Takeichi and Akiyama, 2016). Its major clinical features include congenital hemidysplasia, ichthyosiform nevi, and limb defects. The disease usually presents at birth or shortly thereafter and is frequently associated with abnormalities of other organ systems, such as the nervous and cardiovascular systems (Chen et al., 2025).

Cutaneous manifestations are characterized by ichthyosiform lesions with yellowish, waxy scales, typically without subjective symptoms. The lesions show a unilateral distribution (most commonly on the right side), diffusely involving the trunk while sparing the face, with a sharp midline demarcation or distribution along Blaschko’s lines (Sandov et al., 2019). The NAD(P)H steroid dehydrogenase-like (*NSDHL*, OMIM: 300275) gene encodes a 3β-hydroxysteroid dehydrogenase required for *de novo* cholesterol biosynthesis. This enzyme catalyzes the demethylation of meiosis-activating sterols, leading to the production of zymosterol. Mutations in *NSDHL* result in the accumulation of upstream sterol intermediates, causing impaired cholesterol synthesis and the buildup of toxic metabolites in affected tissues, ultimately giving rise to CHILD syndrome (Kim et al., 2024). As sterols are essential components of cell membranes and myelin sheaths, defects in sterol metabolism can lead to structural abnormalities and functional impairments in the skin and central nervous system. Clinical studies have demonstrated favorable therapeutic outcomes with topical cholesterol combined with statins (Omi et al., 2024).

##### 3.1.2.2 Chondrodysplasia punctata type 2 (CDPX2)

Chondrodysplasia punctata type 2 is an X-linked dominant disorder that predominantly affects females. Its main clinical manifestations include ichthyosis, stippled epiphyses, cataracts, and short stature (Horinouchi et al., 2019). At birth, most affected infants present with generalized erythema, which partially or completely resolves within several weeks after birth.

The *EBP* gene, located downstream of *NSDHL*, encodes Δ8–Δ7 sterol isomerase, an enzyme involved in the *de novo* biosynthesis of cholesterol (Braverman et al., 1999). Mutations in EBP impair cholesterol synthesis and are responsible for the development of CDPX2. Δ8–Δ7 sterol isomerase catalyzes the conversion of zymosterol to dehydrolathosterol during cholesterol biosynthesis. When the activity of this enzyme is compromised, cholesterol production is reduced, leading to decreased cholesterol levels in the skin and subsequent disruption of normal epidermal barrier function and metabolism (Ausavarat et al., 2008). Although CHILD syndrome and CDPX2 share similar clinical features, CDPX2 results from mutations in the *EBP* gene and typically does not exhibit a strictly unilateral distribution of skin lesions, which can aid in differential diagnosis (Jeong et al., 2017).

Both *EBP* and *NSDHL* encode enzymes involved in closely related steps of the cholesterol biosynthetic pathway. However, mutations in these genes give rise to distinct clinical entities, namely, CDPX2 and CHILD syndrome, respectively. This observation suggests that, in addition to cholesterol deficiency, specific sterol intermediates—such as zymosterol and desmosterol—may also play important pathogenic roles. The precise mechanisms underlying their involvement remain to be further elucidated.

##### 3.1.2.3 Palmoplantar keratoderma–congenital alopecia syndrome type 2 (PPKCA2)

Palmoplantar keratoderma–congenital alopecia syndrome type 2 is a rare autosomal recessive keratinization genodermatosis caused by pathogenic variants in the lanosterol synthase (*LSS*) gene, a key component of the cholesterol *de novo* biosynthesis pathway (Xiu et al., 2025). Clinically, the disorder is characterized by progressive palmoplantar hyperkeratosis and congenital or early-onset alopecia, often accompanied by nail abnormalities and, in some cases, extracutaneous manifestations such as ocular or neurological involvement. The combination of prominent cutaneous phenotypes and multisystem features highlights the essential role of sterol metabolism in skin development and homeostasis (Besnard et al., 2019).

LSS encodes lanosterol synthase, the enzyme responsible for catalyzing the conversion of oxidosqualene to lanosterol, which represents the first committed sterol intermediate in cholesterol biosynthesis (Yang et al., 2022). Disruption of LSS function impairs cholesterol production at an early stage of the pathway, leading to sterol imbalance within keratinocytes. Given the critical contribution of cholesterol to lamellar body formation, epidermal lipid organization, and barrier integrity, defects in lanosterol synthesis are thought to compromise terminal keratinocyte differentiation and cornification, ultimately resulting in hyperkeratosis and hair follicle abnormalities (Besnard et al., 2019; Yang et al., 2022).

The clinical phenotype associated with *LSS* mutations further supports the concept that both cholesterol deficiency and accumulation of upstream sterol intermediates can be detrimental to epidermal homeostasis. Similar to other cholesterol-related genodermatoses, such as CHILD syndrome and X-linked ichthyosis, PPKCA2 underscores the importance of tightly regulated cholesterol biosynthesis for normal skin function (Gram et al., 2025; Diociaiuti et al., 2018). Moreover, the phenotypic heterogeneity observed among individuals with *LSS* mutations suggests that residual enzymatic activity, tissue-specific sterol requirements, and differential sensitivity to sterol imbalance may collectively influence disease severity (Yoneda, 2016).

### 3.2 Cholesterol regulatory pathway defects: SREBP-SCAP pathway

The SREBP–SCAP pathway is a sterol-sensing regulatory system that maintains cellular lipid homeostasis. Under low-cholesterol conditions, SREBP is escorted by SCAP from the endoplasmic reticulum to the Golgi for proteolytic activation, enabling transcription of genes involved in cholesterol and fatty acid biosynthesis (Huang et al., 2025). When sterol levels are sufficient, this transport is inhibited, thereby suppressing lipid synthesis through negative feedback control (Zhang et al., 2017).

#### 3.2.1 Ichthyosis follicularis with alopecia and photophobia (IFAP) syndrome

Ichthyosis follicularis with alopecia and photophobia syndrome is a rare form of ichthyosis that can be inherited in an X-linked manner (Murase et al., 2020). The cardinal clinical features include follicular ichthyosis, partial or complete alopecia, and photophobia, and may be accompanied by keratitis, blepharitis, and enamel hypoplasia.

Oeffner et al. reported that mutations in membrane-bound transcription factor peptidase, site 2 (*MBTPS2*, OMIM: 300294) can cause IFAP syndrome (Oeffner et al., 2011), while Wang et al. reported sterol regulatory element binding transcription factor 1 (*SREBF*1, OMIM: 184756) mutations in IFAP-like phenotypes (Wang et al., 2020). S2P is a membrane-embedded zinc metalloprotease that plays a crucial role in activating signaling proteins involved in cholesterol-regulated transcription and the endoplasmic reticulum stress response.

Under normal conditions, the SREBP1–SCAP complex is anchored to the endoplasmic reticulum membrane by INSIG proteins and subsequently transported to the Golgi apparatus, where it undergoes proteolytic cleavage to regulate fatty acid and cholesterol metabolism (Irurzun et al., 2021). S2P not only cleaves SREBP1 but also processes SREBP2, which is primarily responsible for cholesterol homeostasis. Although SREBP1 mainly regulates fatty acid metabolism, its cleaved active fragment can translocate into the nucleus and bind to SREs, thereby upregulating the expression of genes involved in cholesterol biosynthesis (Chen et al., 2022). Therefore, mutations in *MBTPS2* and *SREBF1* may lead to the development of IFAP syndrome by impairing cholesterol metabolic pathways.

#### 3.2.2 Keratosis follicularis spinulosa decalvans (KFSD)

Keratosis follicularis spinulosa decalvans is a rare X-linked genodermatosis characterized by progressive follicular hyperkeratosis, papular lesions, and scarring alopecia, primarily affecting the scalp, eyebrows, and eyelashes (Yoneda, 2016). Patients often present in early childhood with keratotic follicular papules and may develop alopecia that progresses over time, accompanied by ocular abnormalities such as photophobia and corneal dystrophy in some cases. KFSD is caused by mutations in the *MBTPS2* gene, while *CST6* (Cystatin 6, OMIM: 601891) is also reported in KFSD-like phenotypes (Eckl et al., 2021; Fong et al., 2012). Loss-of-function or hypomorphic variants in *MBTPS2* disrupt polyamine homeostasis, leading to abnormal keratinocyte differentiation, follicular plugging, and localized inflammation (Lan et al., 2023). Histopathology typically reveals hyperkeratosis, follicular plugging, perifollicular fibrosis, and chronic inflammatory infiltrates (Lan et al., 2023). The condition exhibits X-linked inheritance, with males typically more severely affected and carrier females showing milder phenotypes due to lyonization. Although KFSD is primarily a cosmetic and functional concern, secondary bacterial infections can exacerbate inflammation (Aten et al., 2010). Therapeutic management is largely symptomatic, including topical keratolytics, retinoids, and anti-inflammatory agents, aiming to reduce follicular hyperkeratosis and preserve residual hair (Zhang et al., 2016). Recent insights into polyamine metabolism suggest that modulation of this pathway could represent a future therapeutic target. KFSD thus provides a valuable model for studying the interplay between keratinocyte differentiation, follicular biology, and metabolic regulation in genodermatoses.

### 3.3 Cholesterol transport and trafficking defects

#### 3.3.1 X-linked ichthyosis (XLI)

X-linked ichthyosis is characterized primarily by dry skin and the presence of large, polygonal, dark brown scales symmetrically distributed over various regions of the body surface (Tang et al., 2022). The disease typically manifests within a few weeks after birth and progressively worsens in both extent and severity. Extracutaneous manifestations, such as corneal opacities and cryptorchidism, are common associated features.

XLI is caused by mutations in the *STS* (OMIM: 300747) gene, with approximately 90% of patients exhibiting complete deletions of the STS gene, which may extend to adjacent genes, resulting in contiguous gene deletion syndromes (Harper et al., 2019; Teng et al., 2022). During the upward transport of cholesterol from the basal layer to the stratum corneum, cholesterol is first converted to cholesterol sulfate by SULT2B1 and subsequently desulfated back to cholesterol by STS.

Loss of STS function leads to the accumulation of cholesterol sulfate, which inhibits corneodesmosome degradation and results in impaired desquamation and scale formation (Zhou et al., 2024). In addition, cholesterol sulfate accumulation suppresses the activity of HMGCR, thereby further impairing cholesterol biosynthesis. Reduced cholesterol content in the stratum corneum compromises epidermal barrier integrity, increases transepidermal water loss, and ultimately leads to skin dryness (Diociaiuti et al., 2018; McGeoghan et al., 2023). Delayed corneodesmosome degradation and prolonged desquamation in XLI contribute to the formation of large, adherent scales on the skin surface.

Recent studies have substantially advanced the understanding of its molecular basis. Notably, RNA sequencing (RNA-seq) analysis of skin tissues from *STS* transgenic and knockout mice provided a global transcriptional landscape of STS deficiency, revealing coordinated dysregulation of pathways governing cell mobility and apoptosis, including Wnt/β-catenin and Hippo signaling (Kwon et al., 2024). These transcriptomic data linked impaired epithelial–mesenchymal transition, elevated ROS production, mitochondrial dysfunction, and activation of Bax/Bak-dependent apoptosis to the XLI phenotype, highlighting RNA-seq as a powerful tool to uncover system-level mechanisms beyond single-gene effects (Kwon et al., 2024). Complementary studies further demonstrated enhanced calcium signaling and keratinocyte differentiation via CasR upregulation, as well as induction of YPEL3-associated cellular senescence in *STS*-deficient models (Kwon et al., 2025; Baek et al., 2021). Together, these findings integrate transcriptomic, signaling, and differentiation abnormalities into a coherent pathogenic framework for XLI.

#### 3.3.2 Multiple sulfatase deficiency (MSD)

Multiple sulfatase deficiency is a rare autosomal recessive form of ichthyosis characterized by multiple congenital anomalies, including a short neck, frontal bossing, kyphosis and/or scoliosis, congenital cartilage calcification, multiple epiphyseal dysplasia, cardiac abnormalities, and laryngeal developmental defects, with ichthyotic skin changes developing progressively over time (Cosma et al., 2003; Dierks et al., 2003).

The protein encoded by *SUMF1* (sulfatase-modifying factor 1, OMIM: 607939) plays a critical role in the conversion of cholesterol sulfate to cholesterol and is involved in cholesterol transport from the basal layer to the stratum corneum (Schlotawa et al., 2020a). The active site of STS contains a highly conserved cysteine residue, which is post-translationally modified to formylglycine under the catalytic action of SUMF1. This modification is essential for STS enzymatic activity in catalyzing the conversion of cholesterol sulfate to cholesterol. Inactivation of SUMF1 results in reduced catalytic activity of STS toward its substrate, thereby inhibiting the conversion of cholesterol sulfate to cholesterol (Schlotawa et al., 2020b; Cappuccio et al., 2020). Although SUMF1 is not directly involved in cholesterol metabolism, sulfatase dysfunction may secondarily affect epidermal lipid homeostasis and barrier integrity.

#### 3.3.3 Harlequin ichthyosis (HI)

Harlequin ichthyosis is the most severe and distinctive subtype of autosomal recessive congenital ichthyosis (ARCI), which is regulated by cholesterol-associated epidermal lipid homeostasis. Affected neonates are often born prematurely and frequently die within days to weeks after birth (Milstone and Choate, 2013). Clinically, HI is characterized by the presence of thick, armor-like hyperkeratotic plates that subsequently fissure to form large yellow scales, accompanied by severe ectropion, eclabium, and auricular deformities (Shibata et al., 2014; Tanahashi et al., 2015).

HI is caused by loss-of-function mutations in the ATP-binding cassette subfamily A member 12 (*ABCA12,* OMIM: 607800) gene, leading to a profound disorder of keratinization (Enjalbert et al., 2020). This condition is thought to result, at least in part, from impaired cholesterol efflux mediated by ABCA12 (Wang et al., 2019; Akiyama et al., 2005). ABCA12 is a member of the ATP-binding cassette transporter superfamily, which hydrolyzes ATP to transport substrates across cellular membranes.

Beyond its well-established role in epidermal ceramide transport, ABCA12 also contributes to cholesterol homeostasis in macrophages. ABCA12 deficiency impairs LXR agonist-induced cholesterol efflux by reducing the abundance and stability of LXRβ, ABCA1 and ABCG1 through a post-transcriptional mechanism. Consequently, macrophages accumulate intracellular cholesterol, exhibit enhanced foam cell formation, impaired reverse cholesterol transport, and accelerated atherosclerosis *in vivo* (Thomas et al., 2009; Akiyama et al., 2006; Fu et al., 2013).

Similar to CHILD syndrome, topical treatment with statins in combination with cholesterol has also been shown to be effective in patients with ARCI, with improvements observed in symptoms such as skin dryness and scaling (Moss et al., 2023; Sitek et al., 2018). These therapeutic responses further support the involvement of cholesterol dysregulation in the pathogenesis of ARCI.

#### 3.3.4 Lamellar ichthyosis (LI)

Lamellar ichthyosis, related to epidermal lipid barrier disorder involving ceramide, acylceramide, and sterol components, is a subtype of ARCI. Clinically, neonates with LI are born encased in a collodion membrane, which typically sheds within approximately 1 week after birth, followed by the gradual development of generalized, large, dark, plate-like scales (Ahogo Kouadio et al., 2017). Erythroderma is mild or absent, and additional features may include ectropion, eclabium, and auricular cartilage dysplasia.

Mutations in multiple genes have been implicated in the pathogenesis of LI, including *ABCA12*, *SULT2B1* (OMIM: 604125), *TGM1* (Transglutaminase 1, OMIM: 190195), *CYP4F22* (Cytochrome P450 family four subfamily F member 22, OMIM: 611495), *ALOXE3* (Arachidonate lipoxygenase 3, OMIM: 607206), *ALOX12B* (Arachidonate 12-lipoxygenase, 12R type, OMIM: 603741), *NIPAL4/ICHTHYIN* (NIPA-like domain containing 4, OMIM: 609383), *SDR9C7* (Short chain dehydrogenase/reductase family 9C member 7, OMIM: 609769), *CERS3* (Ceramide synthase 3, OMIM: 615276), and *PNPLA1* (Patatin-like phospholipase domain containing 1, OMIM: 612121) (Wu and Yao, 2023).

SULT2B1 catalyzes the conversion of cholesterol to cholesterol sulfate in the basal layer of the epidermis. Mutations in *SULT2B1* can lead to cholesterol accumulation and impair cholesterol transport from the basal layer to the stratum corneum (Heinz et al., 2017). The remaining causative genes primarily function in ceramide synthesis. Mutations in these genes disrupt ceramide production and transport, impair epidermal barrier formation, and ultimately lead to the development of LI (De Greef and Baeck, 2024; Nukaly et al., 2025).

Mutations in the *PNPLA1* gene cause autosomal recessive congenital ichthyosis by disrupting epidermal lipid barrier formation (Grall et al., 2012). PNPLA1 is a transacylase expressed in differentiated keratinocytes that catalyzes the transfer of linoleic acid from triglycerides to ω-hydroxyceramides, generating ω-O-acylceramides (acylceramides), which are essential components of the stratum corneum lipid lamellae (Hirabayashi et al., 2017). Loss of *PNPLA1* markedly reduces ω-O-acylceramide production, leading to the near absence of acylceramides and accumulation of precursor lipids (Schratter et al., 2025; Grond et al., 2016). As a consequence, intercellular lipid lamellae are diminished, transepidermal water loss increases, and the epidermal permeability barrier collapses. Proper localization of PNPLA1 to lipid droplets—facilitated by ABHD5—is critical for its enzymatic activity; disease-associated mutations often impair lipid droplet targeting and abolish acylceramide synthesis (Schratter et al., 2025; Nohara et al., 2022). Furthermore, reduced lipid droplet-associated activity limits substrate availability for ω-O-esterification. Collectively, PNPLA1 deficiency disrupts lipid droplet dynamics and acylceramide generation, ultimately compromising skin barrier integrity and leading to ichthyosis.

#### 3.3.5 Congenital ichthyosiform erythroderma (CIE)

Congenital ichthyosiform erythroderma, together with HI and LI, belongs to the spectrum of ARCI, and its clinical presentation overlaps substantially with that of LI (Liu et al., 2025). Newborns with CIE are also encased in a collodion membrane at birth; however, CIE is distinguished by prominent erythroderma and the presence of smaller, whitish scales, which facilitates differentiation from LI (Mazereeuw-Hautier et al., 2025; Mazereeuw-Haut et al., 2025).

Mutations in multiple genes have been identified in CIE, including *ABCA12*, *TGM1*, *CYP4F22*, *ALOXE3*, *ALOX12B*, *CERS3*, *NIPAL4/ICHTHYIN*, *SDR9C7*, *PNPLA1*, and *LIPN* (Lipase family member N, OMIM: 613924) (Heinz et al., 2017; Viedma-Martínez et al., 2022). ABCA12 may participate in epidermal cholesterol metabolism by modulating cholesterol efflux. Notably, *ABCA12* mutations can give rise to both LI and CIE, suggesting that distinct mutation types may differentially affect protein function and metabolic homeostasis, ultimately resulting in divergent clinical phenotypes (van Leersum et al., 2019).

## 4 Therapeutic strategies targeting cholesterol metabolism in genodermatoses of keratinization

Recent advances in understanding the role of cholesterol metabolism in genodermatoses of keratinization have opened new avenues for targeted therapeutic interventions. Given the pivotal functions of cholesterol and its derivatives in maintaining epidermal barrier integrity, keratinocyte differentiation, and desquamation, pharmacological and topical strategies aimed at modulating cholesterol biosynthesis, transport, and metabolism have been explored as potential treatments for inherited ichthyoses and related disorders.

Accumulating evidence indicates that disturbances in cholesterol metabolism may contribute to inflammatory reactions in addition to barrier defects (Bauer et al., 2023). Abnormal accumulation of cholesterol, sterol intermediates, or oxidized cholesterol derivatives (oxysterols) can function as metabolic danger signals that activate innate immune pathways. For example, oxysterols such as 7-Ketocholesterol and 25-Hydroxycholesterol have been shown to promote oxidative stress and stimulate pro-inflammatory signaling in various cell types, including keratinocytes (de Freitas et al., 2022). These molecules can activate transcriptional regulators such as NF-κB, leading to increased expression of inflammatory cytokines including IL-1β, IL-6, and TNF-α. In addition, excessive intracellular cholesterol accumulation may activate the NLRP3 inflammasome, a multiprotein complex that promotes caspase-1 activation and the release of IL-1β and IL-18 (Brahmi et al., 2025). Such inflammasome activation has been linked to metabolic inflammation in several tissues. In the context of lipid metabolism–related skin disorders, toxic sterol intermediates generated by defects in cholesterol biosynthetic enzymes may therefore contribute directly to cutaneous inflammation (Lin et al., 2023; Perryman et al., 2021). These findings suggest that inflammatory signaling triggered by sterol imbalance represents an additional pathogenic mechanism in keratinization disorders, complementing the well-recognized role of epidermal barrier dysfunction.

One promising approach involves the use of topical cholesterol supplementation, particularly in disorders where endogenous cholesterol synthesis or transport is impaired (Paller et al., 2011). In several keratinization disorders, such as CHILD syndrome and certain forms of X-linked ichthyosis, exogenous cholesterol can partially compensate for deficits in epidermal cholesterol, promoting lamellar body formation and restoring stratum corneum lipid composition (Sandov et al., 2019; Alexopoulos and Kakourou, 2015). Topical formulations are often combined with agents that modulate cholesterol biosynthesis, such as statins, to simultaneously reduce the accumulation of cytotoxic sterol intermediates while replenishing functional cholesterol in the epidermis. Statins inhibit HMGCR, the rate-limiting enzyme in *de novo* cholesterol synthesis, thereby preventing the buildup of toxic metabolites upstream in the cholesterol biosynthetic pathway (Kallis et al., 2021). Clinical studies have demonstrated that this combination therapy can lead to significant improvement in skin dryness, scaling, and overall barrier function in affected patients (Khalil et al., 2018).

In addition to statins, other small-molecule inhibitors and enzyme modulators targeting specific steps of cholesterol metabolism are under investigation. For example, interventions aimed at modulating SREBP–SCAP signaling have potential therapeutic relevance, as dysregulation of this pathway can result in altered transcription of cholesterol biosynthesis genes and impaired keratinocyte lipid homeostasis (Eberlé et al., 2004). Pharmacological agents that normalize SREBP processing could restore cholesterol synthesis and uptake, thereby improving stratum corneum integrity in patients with SREBP-related genodermatoses. Similarly, therapies targeting the HMGCR degradation pathway may provide a means to fine-tune cholesterol levels in keratinocytes, avoiding both cholesterol deficiency and toxic accumulation of sterol intermediates.

The cholesterol transport pathway within the epidermis also represents a viable therapeutic target. Enzymes such as SULT2B1 and STS regulate the conversion between cholesterol and cholesterol sulfate, which is crucial for corneodesmosome turnover, desquamation, and barrier function (Heinz et al., 2017). Deficiencies in these enzymes lead to either accumulation or depletion of cholesterol sulfate, resulting in impaired desquamation and pathological scaling (Elias et al., 2013). Therapeutic strategies that restore the balance between cholesterol and cholesterol sulfate, either through topical or pathway-level modulation strategies are under investigation.

In addition to pharmacological approaches, gene therapy and molecular correction strategies are emerging as potential long-term solutions for cholesterol-related keratinization disorders. Targeted correction of mutations in genes encoding cholesterol biosynthesis enzymes, transporters, or modifiers (e.g., NSDHL, EBP, ABCA12) could restore normal cholesterol metabolism in keratinocytes. Early preclinical studies using viral vectors or clustered regularly interspaced short palindromic repeats (CRISPR)-based approaches have demonstrated the feasibility of gene correction in *ex vivo* keratinocyte models.

Emerging therapeutic strategies are expanding beyond conventional topical and systemic treatments for inherited keratinization disorders associated with lipid metabolism defects (Dairov et al., 2025). Among these, investigational stem cell–based therapies have attracted increasing attention due to their regenerative and immunomodulatory potential. Mesenchymal stem cells (MSCs) and induced pluripotent stem cell (iPSC)-derived keratinocytes may restore epidermal homeostasis by promoting tissue repair, modulating inflammatory responses, and potentially correcting metabolic abnormalities in epidermal lipid synthesis (Khalilzad et al., 2024). In parallel, extracellular vesicles, particularly exosomes, have emerged as promising cell-free therapeutic tools. Exosomes derived from stem cells or keratinocytes can transfer lipids, enzymes, and regulatory RNAs that influence cellular metabolism and signaling (da Costa Pereira Cestari et al., 2025). Recent studies suggest that exosome-mediated delivery of metabolic regulators could modulate lipid biosynthesis pathways in keratinocytes, thereby improving epidermal barrier function (Pham, 2026). This approach may be particularly relevant for lipid metabolism–related genodermatoses, where defects in ceramide, cholesterol, or fatty acid pathways disrupt the stratum corneum lipid matrix.

Another emerging concept linking lipid metabolism and skin homeostasis is efferocytosis, the process by which apoptotic cells are cleared by phagocytes. Efficient efferocytosis not only prevents chronic inflammation but also contributes to lipid recycling and metabolic regulation within tissues (Wang et al., 2024). In the skin, impaired clearance of dying keratinocytes may alter local lipid handling and inflammatory signaling, thereby exacerbating barrier dysfunction. Understanding how efferocytosis intersects with epidermal lipid metabolism could reveal new therapeutic targets for disorders characterized by abnormal keratinization and lipid imbalance (Yan et al., 2025). Together, advances in stem cell biology, exosome-based delivery systems, and efferocytosis research provide promising avenues for developing innovative treatments that address both the metabolic and structural aspects of lipid metabolism–associated skin diseases.

Moreover, combination strategies integrating topical cholesterol supplementation, metabolic enzyme modulation, and gene therapy hold promise for synergistic effects. Personalized treatment approaches based on the specific gene defect and the nature of cholesterol metabolic disruption may optimize therapeutic efficacy while minimizing adverse effects (Elias et al., 2012).

Overall, therapeutic interventions targeting cholesterol metabolism represent a rational and increasingly evidence-based approach to managing genodermatoses of keratinization. Continued research into the precise molecular mechanisms underlying cholesterol dysregulation in the epidermis, coupled with advances in pharmacology and gene therapy, is likely to yield more effective and targeted treatments. The combination of mechanistic insight and translational research provides a strong foundation for developing strategies that not only alleviate symptoms but also address the underlying metabolic defects, offering hope for durable improvement in patients with inherited disorders of keratinization.

## 5 Conclusions and perspectives

Cholesterol and its metabolites play pivotal roles in the formation of epidermal lamellar bodies and in maintaining the structural integrity of the permeability barrier. Disruptions at any stage of cholesterol synthesis, transport, modification, or degradation can interfere with normal lamellar body biogenesis and lipid lamellae organization, thereby contributing to the pathogenesis of genedermatoses. Importantly, accumulating evidence indicates that cholesterol biosynthesis disorders should not be viewed solely as conditions of structural lipid deficiency, but rather as complex metabolic diseases in which altered sterol composition reshapes keratinocyte signaling, differentiation, and inflammatory responsiveness. At present, the causative genes remain unidentified in a subset of patients with genedermatoses. Given the intimate connection between epidermal lipid homeostasis and sterol metabolism, it is plausible that yet-unrecognized regulators of cholesterol biosynthesis, sterol trafficking, or sterol sensing may underlie disease in these individuals.

Notably, the causative genes of many genodermatoses subtypes include not only those directly involved in cholesterol metabolism but also genes encoding structural, barrier-related proteins, lipid transporters, and barrier-associated molecules. How mutations in these distinct functional categories converge to produceoverlapping clinical phenotypes remains an unresolved question. A pathway-oriented perspective suggests that diverse genetic defects may ultimately converge on common downstream mechanisms, including disrupted sterol homeostasis, altered membrane microdomain organization, and secondary activation of innate immune pathways. Furthermore, mutations in a single gene can lead to multiple ichthyosis subtypes—for example, allelic and functional variability of *ABCA12* contributes to phenotypic spectrum—underscoring the need to elucidate how quantitative differences in lipid imbalance, sterol intermediate accumulation, and inflammatory signaling contribute to genotype–phenotype variability.

Of particular interest, combined topical therapy with statins and cholesterol has shown favorable therapeutic effects in certain ichthyosis patients. These observations provide clinical support for a dual-pathogenic model in which toxic sterol precursor accumulation, rather than cholesterol deficiency alone, plays a central role in disease pathogenesis. Given that more than thirty intermediate metabolites are generated during cholesterol biosynthesis, identifying the key pathogenic intermediates will be of great significance for the development of future targeted therapeutic strategies. Mechanistically stratified therapeutic approaches targeting cholesterol restoration, reduction of toxic sterol load, or modulation of metabolic–immune signaling interfaces may ultimately enable more precise and effective treatment strategies.

Several limitations should be acknowledged. First, many cholesterol-associated genodermatoses are ultra-rare disorders with limited mechanistic and clinical data available. Second, much of the current evidence is derived from case reports, animal studies, or *in vitro* experiments, limiting generalizability. Third, publication bias toward genetically characterized or severe phenotypes may influence current understanding. Finally, although this review discusses emerging therapeutic strategies, many approaches remain investigational and lack clinical validation.

In summary, advancing our understanding of how sterol metabolism integrates epidermal barrier biology with immune regulation will be essential for redefining cholesterol-related ichthyosis as a spectrum of metabolic–inflammatory disorders. Future research should focus on clarifying the identity and signaling properties of pathogenic sterol intermediates, delineating cell-type–specific metabolic regulation within the epidermis, and exploring targeted interventions at the metabolic–immune interface.
